# The Use of Text Messaging to Improve Clinical Engagement for Individuals With Psychosis: Systematic Review

**DOI:** 10.2196/16993

**Published:** 2020-04-02

**Authors:** Jessica D'Arcey, Joanna Collaton, Nicole Kozloff, Aristotle N Voineskos, Sean A Kidd, George Foussias

**Affiliations:** 1 Slaight Centre for Youth in Transition Centre for Addiction and Mental Health Toronto, ON Canada; 2 Institute of Medial Science University of Toronto Toronto, ON Canada; 3 Department of Clinical Psychology University of Guelph Guelph, ON Canada; 4 Institute for Health Policy Management and Evaluation University of Toronto Toronto, ON Canada; 5 Department of Psychiatry Centre for Addiction and Mental Health Toronto, ON Canada

**Keywords:** SMS, text messaging, psychosis, schizophrenia, bipolar disorder, engagement, medication adherence, attendance, patient appointments

## Abstract

**Background:**

Individuals experiencing psychosis are at a disproportionate risk for premature disengagement from clinical treatment. Barriers to clinical engagement typically result from funding constraints causing limited access to and flexibility in services. Digital strategies, such as SMS text messaging, offer a low-cost alternative to potentially improve engagement. However, little is known about the efficacy of SMS text messaging in psychosis.

**Objective:**

This review aimed to address this gap, providing insights into the relationship between SMS text messaging and clinical engagement in the treatment of psychosis.

**Methods:**

Studies examining SMS text messaging as an engagement strategy in the treatment of psychosis were reviewed. Included studies were published from the year 2000 onward in the English language, with no methodological restrictions, and were identified using 3 core databases and gray literature sources.

**Results:**

Of the 233 studies extracted, 15 were eligible for inclusion. Most studies demonstrated the positive effects of SMS text messaging on dimensions of engagement such as medication adherence, clinic attendance, and therapeutic alliance. Studies examining the feasibility of SMS text messaging interventions found that they are safe, easy to use, and positively received.

**Conclusions:**

Overall, SMS text messaging is a low-cost, practical method of improving engagement in the treatment of psychosis, although efficacy may vary by symptomology and personal characteristics. Cost-effectiveness and safety considerations were not adequately examined in the studies included. Future studies should consider personalizing SMS text messaging interventions and include cost and safety analyses to appraise readiness for implementation.

## Introduction

### Psychosis and Engagement

The major psychoses (ie, schizophrenia spectrum and bipolar I disorder) are recognized as a leading cause of disability worldwide [1] and are associated with poverty [2], premature mortality [3], impaired cognitive function [4], loss of education and employment [5], and increased global economic burden [6]. Published practice guidelines outlining the usual treatment for psychosis identify antipsychotic medication as a frontline treatment for positive symptom management and adjunct psychosocial interventions such as psychoeducation, family support, vocational interventions, and cognitive behavioral skills–based therapies [7-11]. However, despite concerted treatment efforts, disengagement from clinical services remains to be a significant barrier to recovery for this population as evidenced by high rates of nonadherence (25%-50%) [12], nonattendance (40%) [13], and service dropout (30%) [14].

### Background

Clinical engagement is a combination of health-oriented attitudes (ie, beliefs about the cause of illness and intentions of the care team) and behaviors (ie, appointment attendance, adherence to treatment plans, and the level of participation in a clinical relationship) [15,16]. Therefore, disengagement occurs when attitudinal factors cause patients to stop performing treatment behaviors such as taking medications and attending appointments [16]. Disengaged individuals often exhibit greater functional impairment and higher symptom burden over time [17,18], subsequently leading to higher rates of relapse and rehospitalization [12,13]. Cycles of relapse and rehospitalization create a unique conundrum for this population as evidence suggests that with each period of disengagement and relapse, positive symptoms become more severe and more difficult to treat [19]. This group is also less likely to fully recover or hold employment [18]. Moreover, studies show that early and sustained engagement in treatment can mitigate negative outcomes of psychosis such as significantly lowering all-cause mortality [20], reducing the risk of relapse [7-11] and ameliorating functional impairment [5,9]. As such, understanding and improving engagement in this population is a priority.

Barriers to clinical engagement are rooted in access to treatment including geographic, systemic, and financial obstacles. Psychosocial services are often located in urbanized areas, serving specific catchment areas [21] and limiting access for rural or remote residents [15,21-23]. Moreover, services are often only available in a business model (ie, Monday-Friday, 9 AM-5 PM) [24-26], with clinicians often treating double the recommended caseloads, further restricting appointment availability [26] because of funding constraints [24-26]. These challenges are often exacerbated by personal factors such as poverty, limited transportation, substance use, trauma history, and beliefs about mental health [15].

Given the financial and staff limitations, there has been a call for more cost-effective, flexible modes of engagement, such as SMS text messaging. SMS text messaging is a globally used mechanism for communication across both wealthy and poor countries [27], and cellular phone ownership and its use in populations with a psychiatric illness is high (72%-97%) [28-31]. SMS text messaging offers an easy, user-friendly platform to extend opportunities for engagement beyond clinical boundaries. SMS text messaging also does not require access to a smartphone, wireless internet, or cellular data, making it a more accessible platform for low-income populations, including those with psychosis. These characteristics may allow SMS text messaging to provide support remotely, and in real time, to boost engagement as shown in other areas of health care such as HIV [32], diabetes [33-35], coronary heart disease [36], obesity [37], and substance use disorders [38,39].

### This Study

This systematic review aimed to examine studies investigating SMS text messaging strategies to improve clinical engagement in individuals with psychosis. To date, reviews have focused on the subdomains of clinical engagement such as clinic attendance or medication adherence; however, no such review or single study has examined engagement holistically nor have reviews focused on the use of SMS text messaging within populations with psychosis. This is an important knowledge gap given the challenging nature of engagement in the treatment of psychosis, the resulting considerable outcome disparities, and recent trends toward technologically aided health care. As such, even a modest improvement in clinical engagement could lead to better cost-effectiveness of psychosis treatments [40].

## Methods

### Study Inclusion Criteria

Studies were included regardless of the methodological framework or quality, patient setting (ie, inpatient, outpatient, or community care), age of the population, or stage of illness to provide an inclusive examination of clinical engagement. Studies with a range of quality ratings were included to fully examine the state of the literature on this topic. Included interventions used SMS text messaging as a delivery platform, whereas interventions with mixed or non–SMS text messaging technology (eg, mobile apps) were excluded. This review focused on treatment-seeking individuals with psychosis (ie, the portion of the population either beginning or receiving treatment) to isolate the effect of SMS text messaging on clinical engagement. Although it is true that disengagement is a challenge for those who have yet to be in contact with the mental health care system, attempting to study this population would shift focus from treatment engagement to treatment outreach.

### Primary Outcomes: Clinical Engagement

Clinical engagement is a broad term describing patients’ participation in treatment and is typically measured using its behavioral outcomes such as appointment attendance (eg, number or percentage of appointments attended, cancelled, or rescheduled), medication adherence (eg, pill counts, clinician or self-estimates, pharmacy prescriptions, or blood plasma levels), and service dropout (eg, complete disengagement from care). However, the consideration of behavioral outcomes alone does not provide a holistic examination of clinical engagement as it excludes the attitudinal aspects of this complex and nuanced concept. To this end, we also included studies with outcomes measuring attitudes toward medication, the level of involvement or participation in treatment plans, and therapeutic rapport.

### Secondary Outcomes: Feasibility

Feasibility studies were also included to assess 2 outcomes: the level of patient engagement with the SMS text messaging intervention itself, and the degree of practicality of SMS text messaging interventions and their ease of implementation. Outcomes of feasibility trials include user feedback on ease of use, satisfaction, likelihood of future use, and overall intervention design. Outcomes related to practicality and implementation include associated risk and economic, technical, and legal considerations. These are important outcomes to consider when assessing the efficacy of interventions because both directly affect the interventions’ clinical utility. An intervention of any kind is only as successful as its ability to engage its users. In this case, engagement in the SMS text messaging interventions aims to improve engagement in the treatment of psychosis. Thus, this review examined both aspects of engagement to comprehensively evaluate the efficacy and clinical utility of SMS text messaging interventions to improve clinical engagement in populations with psychosis.

### Search Strategy

Web-based databases cataloging meta-analyses, systematic reviews, and protocols were searched to ensure the originality of this protocol (ie, Cochrane and PROSPERO); however, there were no results on existing reviews on the use of SMS text messaging in psychosis. A unique protocol was, then, created following the Preferred Reporting Items for Systematic reviews and Meta-Analyses guidelines and in consult with a librarian at the University of Toronto. The resulting protocol was registered with PROSPERO (reference number: CRD42018091962), an international protocol registry for prospective reviews in health care managed by the National Institute of Health Research.

In all, 3 core databases powered by OVID were used: PsycINFO, MEDLINE (Medical Literature Analysis and Retrieval System Online), and EMBASE, using the following search strategy: schizophrenia spectrum.mp. OR psychotic disorder.mp. OR exp psychosis/ OR exp bipolar disorder/AND (sms or short messag* service* or texting or text messag*).mp. Please note that .*mp*. is a mapping command that allows you to search using a word or phrase across titles, keywords, and abstracts using databases powered by OVID.

CINAHL and Google Scholar were used as peripheral databases to ensure studies were not missed. Search terms and keywords related to psychosis and SMS text messaging that were used to search gray literature included the following: (1) SMS OR Short Message Service OR SMS-Survey OR Texting OR Text Message OR SMS Based System OR SMS Reminder OR Text Message Reminder and (2) Psychosis OR Schizophrenia OR Bipolar OR Schizoaffective OR Schizophrenia Spectrum OR Psychotic Disorders OR First-Episode Psychosis OR Early-Episode Psychosis. Additionally, reference lists of identified studies were hand searched.

The search criteria were limited to studies published in the English language, restricting publication dates from January 2000 to March 2019 as there were no SMS text messaging intervention studies published before the year 2000.

### Review Process

Studies were independently reviewed by title, abstract, or full-text using a Web-based blinded review platform named Covidence, which allows the reviewers to log-in independently to conduct their review and tracks conflicts to be resolved. Conflicts were resolved first by the 2 primary reviewers, authors JD and JC, and then by a third reviewer, GF, if necessary. Only full-length empirical studies were reviewed; conference abstracts and commentaries were excluded given their limited descriptions of methodologies and inability to be adequately appraised.

### Data Extracted

We extracted sample sizes, and demographic characteristics including age and gender, and diagnoses. Methods were also extracted, including the study design, intervention type, primary and secondary outcome variables, and statistical plan. To better understand the significance of the results, effect sizes were also extracted from studies with randomized controlled trial (RCT) and quasi-experimental designs and converted into a comparable measure of effect size, Cohen *d*. Finally, results reported in each study were extracted, including primary and secondary results (including *P* values, if available) as well as any other findings of interest to the topic of the review.

### Quality Appraisal

As studies were included regardless of the study design, 3 checklists from the Joanna Briggs toolkit were used: RCTs, quasi-experimental designs, and qualitative studies [41]. This toolkit is an open-access, widely used appraisal tool for systematic reviews accessible through the citation provided. Using the checklist that corresponded to the study type, studies received 1 point per criterion met. Points were tallied and converted to a percentage based on the total number of criteria outlaid on the checklist used for easy comparison across study designs. However, results were compared and contrasted based on the matching outcome variable and study design.

## Results

**Figure 1:**
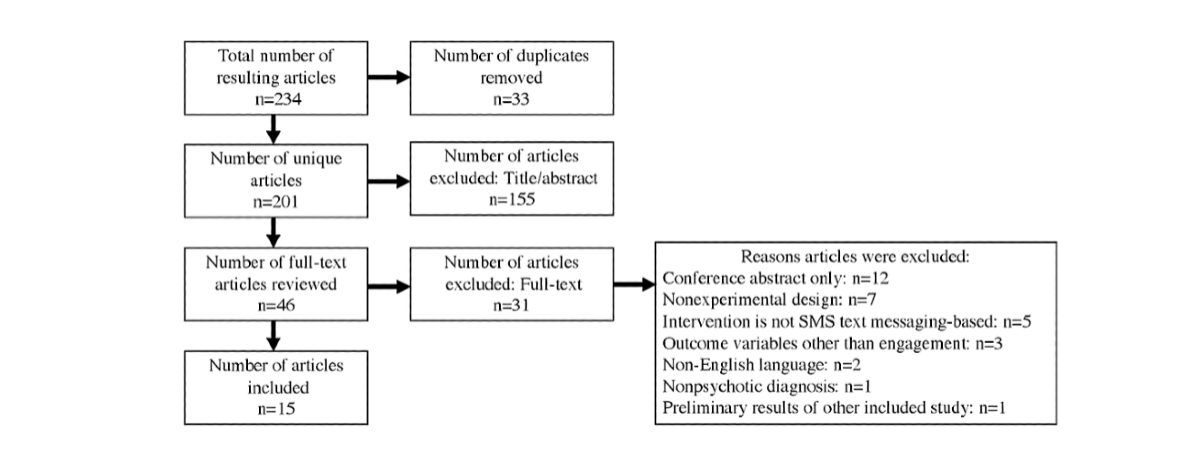
Flow chart of search results.

The original searches of databases yielded 234 studies (Figure 1). The removal of duplicates, as well as a review of titles and abstracts, led to the exclusion of 188 studies. A subsequent full-text review of the remaining 46 articles led to the exclusion of an additional 31 articles, resulting in 15 studies meeting the criteria for inclusion in this review: 8 RCTs, 1 quasi-experimental design, and 6 qualitative studies.

### Study Characteristics

As outlined in Table 1, studies were conducted in a range of countries, although most were Western, developed countries from Europe and North America, with 1 study each from Nigeria, China, and India. They were published from 2010 to 2018, with steady publication rates throughout. Most studies included patients with a diagnosis of schizophrenia spectrum disorders, aged 18 years and older, with 1 study focusing on a younger population, aged 18 to 29 years. Sample sizes ranged from 17 to 1139 with some studies based on the same sample but reporting on different outcomes (eg, studies by Ben-Zeev [42] and Aschbrenner et al [43]; and studies by Kauppi et al [44], Kannisto et al [45], and Välimäki et al [46]). RCTs tended to examine the effects of SMS text messaging reminders on medication adherence and/or clinic attendance paired with secondary outcomes related to symptom severity and functioning. Qualitative studies examined the feasibility and acceptability of SMS text messaging interventions.

**Table 1:** Characteristics of the studies included.

References	Year	Country	Diagnosis/population	Values, n	Age (years), range (mean)	Male:Female
Välimäki et al [46]	2017	Finland	Psychosis	1139^a^	18-65 (38.3)	1:2
Kauppi et al [44]	2015	Finland	Psychosis	562^a^	18-65 (38.6)	1:1
Kannisto et al [45]	2015	Finland	Psychosis	403^a^	18-65 (39.7)	1:1
Montes et al [47]	2012	Spain	SZ^b^	340	18-65 (39.6)	2:1
Xu [48]	2017	China	SZ	237	35-60 (45)	1:1
Menon et al [49]	2018	India	Bipolar I disorder	132	18-65 (37.9)	1:1
Beebe et al [50]	2014	United States	SZ and SZA^c^	30	21-68 (48.7)	1:2
Thomas et al [51]	2017	Nigeria	Psychosis	192	18-64 (33.7)	1:1
Pijnenborg et al [52]	2010	Netherlands	SZ spectrum	62	NR^d^ (28)	4:1
Kravarti et al [53]	2018	United Kingdom	SZ spectrum	75	NR (42.14)	1:1
Granholm et al [54]	2012	United States	SZ and SZA	55	18 (48)	2:1
Ben-Zeev et al [42]	2014	United States	SZ, SZA, and SU^e^	28^f^	18 (40.5)	2:1
Aschbrenner et al [43]	2016	United States	SZ, SZA, and SU	17^f^	18 (NR)	NR
Lal et al [55]	2015	Canada	Early psychosis	67	18-35 (25.6)	3:1
Bogart et al [56]	2014	United Kingdom	Antipsychotic use	85	18-67 (NR)	1:1

^a^Indicates a shared sample.

^b^SZ: schizophrenia.

^c^SZA: schizoaffective disorder.

^d^NR: not reported.

^e^SU: substance use.

^f^Indicates a shared sample.

### Appraisal of Study Quality

Studies included in this review were of a reasonable quality (range 31%-85%); however, no study met all of the criteria laid out in the Joanna Briggs frameworks. Individual study ratings are included in Table 2. RCTs received a range of scores from 31% to 85%, with sample sizes between 28 and 1119 participants. Common unmet criteria in RCTs were blinding expectations, as none of the included trials were double-blinded and many failed to use a concealed assignment. Additionally, some trials were underpowered for their results. In the quasi-experimental study, there was no control group used, causing multiple criteria to be unmet. Qualitative designs tended to satisfy a higher proportion of the quality criteria, receiving scores from 40% to 80% with sample sizes ranging from 17 to 562. These studies often lacked information around experimenter bias and sampling methods and an adequate representation of the population. Of note, feasibility studies do not fit smoothly within the qualitative study category and, therefore, appraisal was limited in this domain. Despite these methodological limitations, these studies provide adequate assessments of and insights into a variety of outcomes related to SMS text messaging, clinical engagement, and the consideration of SMS text messaging implementations.

The quality of outcomes within the included studies was fair, although heterogeneous. The included studies showed inconsistent methods of measurement across medication adherence, often relying on self-report, and the calculation of attendance rates was unclear. Other considerations included small sample sizes as 4 of the experimental studies had samples under 100, and only 1 trial had insufficient power to detect a significant difference [50]. The resulting effect sizes were also variable, ranging from moderate to high. Additionally, the intervention design was inconsistent from the type of SMS text messaging (ie, one-way vs two-way messaging) to the frequency, length, and content of the messages themselves. Only 2 of the included studies were designed to include a follow-up period, both examining participants’ adherence up to 3 months postintervention [47,49]. Taken together, this amount of variance makes it difficult to assess the overall success of the included interventions; however, no clear patterns emerge to suggest that sample size, message type, or effect size varies in response to any methodological factors in these trials. Individual study intervention details are outlined in Table 2 and procedures are outlined in Table 3. 

A summary of the main results has been provided in Multimedia Appendix 1.

**Table 2:** SMS text messaging intervention details of the studies included that performed a trial.

References	Study setting	Reminder type	Frequency of SMS text messaging	Length	Follow-up?	Compensation	Delivery platform	Automated vs Manual	One- vs two-way messaging
Välimäki et al [46], Kannisto et al [45], Kauppi et al [44]^a^	Inpatient discharge	SMS text messaging	2 to 12 messages per month based on the participant	1 year	No	NR^b^	Web platform	Semiautomated	One-way
Montes et al [47]	Outpatient/Community	SMS text messaging	Daily	3 months	Yes, 3 months post	NR	Web platform	Automated	One-way
Xu [48]	Rural community	SMS text messaging to the patient/lay health support person	Up to 2 messages per day based on the participant	6 months	No	Points per response in exchange for hygiene items	Web platform	Semiautomated	Two-way
Menon et al [49]	Outpatient	SMS	2 messages per week	3 months	Yes, 3 months post	NR	Cell phone	Manual (study principal investigator)	One-way
Beebe et al [50]	Community	SMS text messaging and a call	Daily	3 months	No	US $10 per monthly assessment	Cell phone	Manual (mental health nurse)	Two-way
Thomas et al [51]	Outpatient	SMS text messaging	5 and 3 days before the appointment	5 days	No	NR	Web platform	Automated	One-way
Pijnenborg [52]	Outpatient	SMS text messaging	1 message per week	7 weeks	No	NR	Web platform	NR	One-way
Kravariti [53]	Community	SMS text messaging	7 days and 1 day before the appointment	6 months	No	Not paid	Web platform	Automated	One-way
Granholm et al [54]	Outpatient	SMS text messaging	12 messages per day	3 months	No	US $35 for in-person assessments, US $20 for text message-based assessments	Web platform	Automated	Two-way
Been-Zeev et al [42], Aschbrenner et al [43]^a^	Community	SMS text messaging vs calls	Up to 3 messages per day based on the preference	12 weeks	No	Reimbursed up to EUR 30 per month	Cell phone	Manual (social worker)	Two-way

^a^Indicates a shared sample.

^b^NR: not reported*.*

**Table 3:** Methodological characteristics of the study design and methods in the included studies.

References		Study design	Engagement target	Primary outcome	Measurement	Secondary outcomes		Analysis	Quality rating (%)	
**Studies based on intervention trials**										
	Välimäki [46]^a^	RCT^b^ 1:1 (TAU^c^)	MedAd^d^	Number of Hospitalizations	Chart review	Admission type, quality of life, and user satisfaction		OR^e^ and risk ratio	62	
	Montes et al [47]	RCT 1:1 (TAU)	MedAd	MedAd	Self-report	Symptoms, insight, quality of life, and treatment attitude		Stepwise linear regression	69	
	Xu [48]	RCT 1:1 (WC^f^)	MedAd and AppAttd^g^	MedAd and AppAttd	Pill count and scripts	Symptoms and functioning		Generalized estimating equation	85	
	Menon et al [49]	RCT 1:1 (WC)	MedAd and treatment attitude	MedAd	Self-report	Symptoms and quality of life		Repeated measures ANOVA^h^	77	
	Beebe et al [50]	RCT 1:1:1^i^ (NC)	MedAd	MedAd	Pill count	Symptoms		ANOVA	54	
	Thomas et al [51]	RCT 1:1 (TAU)	Initial AppAttd	Initial AppAttd	Attendance dichotomous variable (Yes or No)	Duration of untreated psychosis and symptoms		OR	69	
	Pijnenborg et al [52]	RCT 1:1 (WC)	AppAttd	Number of goals attained (including AppAttd)	Number of appointments attended	Role functioning, symptoms, cognition, and treatment attitude		Multiple linear regression	31	
	Kravariti et al [53]	RCT 1:1 (TAU)	AppAttd	AppAttd	Number of appointments Attended	N/A^j^		Proportions (%), OR	62	
	Granholm et al [54]	Quasi-Experimental (NCon)	MedAd	MedAd, symptoms, and socialization	Ambulatory monitoring	Role functioning and cognition		HGLM^k^	55	
**Studies based on feasibility trials**										
	Ben-Zeev et al [42]^a^	Feasibility	TxAll	User engagement	Self-report	User feedback		Proportions (%) and paired *t* test	80	
	Aschbrenner et al [43]^a^	Qualitative	Reminder interest	User interest	Thematic coding of SMS text messages	N/A		Thematic analysis	70	
	Lal et al [55]	Feasibility	Preferred platform	User interest	Survey	N/A		Proportions (%)	80	
	Bogart et al [56]	Feasibility (survey)	MedAd	User feedback	Self-report	N/A		Proportions (%) and stepwise multilinear regression	40	
	Kauppi et al [44]^a^	Feasibility	MedAd and AppAttd	Preferred topic	Patient message selection	Preferred timing		Proportions (%)	60	
	Kannisto et al [45]^a^	Feasibility	MedAd	User feedback	Survey	Preferred topic and platform		Proportions (%)	62	

^a^Indicates a shared sample.

^b^RCT: randomized controlled trial.

^c^TAU: treatment as usual.

^d^MedAd: medication adherence.

^e^OR: odds ratio.

^f^WC: waitlist control.

^g^AppAttd: appointment adherence.

^h^ANOVA: analysis of variance.

^i^Group allocation for this study is daily SMS text messaging only, weekly phone calls only, and a combined group (daily SMS text messaging and weekly phone calls)*.*

^j^N/A: not applicable.

^k^HGLM: Hierarchical General Linear Modelling.

### Primary Outcome: Clinical Engagement

#### Medication Adherence

In total, 6 of the 8 experimental studies were aimed specifically at improvement of medication adherence, with most studies reporting moderate improvements [49-52]; 2 studies reported on the effect of SMS text messaging medication reminders on participatns attitude toward medication, which showed significant improvement over the course of the intervention [47,48]; 2 other studies reported on a follow-up period and found evidence of potential maintenance effects 3 months after the cessation of the SMS text messaging intervention [47,49]; and 1 study did not report positive findings using hospitalization rates as a function of medication adherence as their primary outcome [46].

In total, 3 studies reported a nonsignificant positive change in the overall group that was significant in subgroups. Specifically, efficacy was influenced by baseline adherence and participants' type of living environment such that those with low baseline adherence [48], or those living independently [54], showed significant improvement while individuals with high baseline adherence [48] or those living with a support person [48,54] showed stable and unchanged adherence rates. Additionally, 1 study even included support persons in the design of the SMS text messaging intervention to receive back up SMS text messaging reminders in the event that participants did not respond [48]. This study shows a positive change in adherence, although, unfortunately, it is not clear how much involvement was required from the individual in the support role [48].

An effectiveness trial compared weekly phone calls with a mental health nurse to daily SMS text messaging medication reminders and a combination group [50]. This study found that phone and SMS text messaging reminders showed the most improvement, followed by the phone only group, then SMS text messaging only [50]. This suggests that adherence also may be boosted with the addition of weekly clinician calls. Participants who were prescribed depot medications were included in this study and were unevenly distributed among the treatment groups (phone+SMS text messaging= 3; SMS text messaging only= 5; and phone only=7), which may bias adherence rates favorably for the phone only group [50].

One of the qualitative studies described underlying reasons for nonadherence. Patients reported that a large proportion (49%) of missed doses were purposeful, rather than owing to forgetfulness (35%) [56]. The most common reasons for intentionally skipping doses were as follows: side effects, attenuated symptoms, and thinking a lower dose would be more beneficial [56]. Negative attitudes toward medication, insufficient information about medication, and being male led to a 3 to 4 times greater likelihood of reporting purposely skipping doses of medication [56].

#### Appointment Attendance and Therapeutic Rapport

In total, 3 studies examined the efficacy of SMS text messaging reminders to improve appointment attendance. Studies demonstrated that SMS text messaging interventions are successful in increasing clinic attendance. SMS text messaging reminders increased the likelihood of attending initial appointments [51] and general attendance during the course of the intervention [52,53]. Studies comparing patient-reported rapport ratings of their usual clinical care teams, and their research-based SMS text messaging interventionist found that, typically, the SMS text messaging interventionist was rated more favorably [42,48]. This is interesting given the difference in modality of the relationship, one based in-person and the other purely electronic and text based.

### Secondary Outcome: Feasibility

#### Practical Considerations

**Figure 2:**
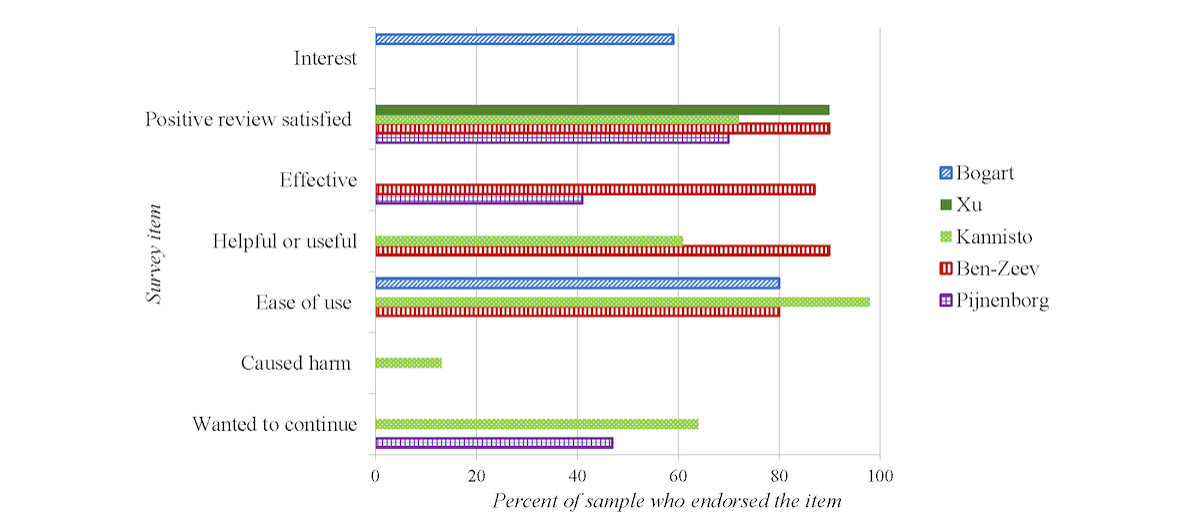
Feasibility data collected from included studies.

In total, 5 studies, including both feasibility designs and experimental designs, examined aspects of feasibility, usability, and user satisfaction as outlined in Figure 2. Studies reported a good endorsement of interest (59%) [56], wanting to continue using the intervention (47%-64%) [42,45,52], moderate-to-high ratings of effectiveness (41%-87%) [42,52], satisfaction (70%-90%) [42,45,48,52], and ease of use (80%-98%) [42,45,56]; only a small proportion endorsed harm associated with the intervention (13%) [45]. Sources of harm included participants finding the SMS text messaging reminder to be disruptive to sleep or work, or annoying [45]. Additionally, 2 studies reported on mobile phone ownership, which was high, ranging from 82% to 94% [55,56]. Other practical considerations include legal implications (eg, privacy and personal health information), work load, and cost-effectiveness. One study reported on cost, which estimated the total cost of the texting intervention from a technological and staff perspective but did not analyze how the intervention changed the overall cost in the clinic or if it was cost-efficient [46]. Another study highlighted concerns and considerations around privacy and risks associated with loss, theft, or disposal of mobile phones containing health information, recommending organizations to perform a detailed risk analysis of network security and processes for ensuring informed consent; however, no specific recommendations were made, and privacy was not surveyed or examined in the trial design [42].

#### Engagement With the SMS Text Messaging Intervention

The success of the interventions depended largely on the patients’ level of participation and response. In all, 3 studies divided their samples using a participation threshold differentiating who tended to respond to SMS text messages and who did not [47,52,54]. The subgroup that tended not to respond to messages showed several demographic and baseline clinical differences. This group was younger, consisted of males, not working or in school, and younger at first contact with treatment [45] and were on polar ends of the clinical and functional spectrum [47,52,54]. In a minority of patients, inpatient hospitalization was reported as a barrier to answering SMS text messages as access to mobile devices is sometimes limited [42]. In the group that did tend to respond to SMS text messages, response rates were high, ranging from 85% to 87% [42,54] regardless of the message topic [54].

Generally, study attrition rates were low, ranging from 0% to 20% (see Table 2). Barriers to using SMS text messaging reported by participants included the following: 22% reported no interest in using such platforms in care, 20% reported lack of time, and 30% reported no barriers; only 7.5% reported lack of device (cell phone) as a barrier [55]. Limitations in understanding engagement with SMS text messaging are centered around the inability to confirm whether or not patients actually received the SMS text messaging reminder in the absence of a response; thus, one-way SMS text messaging cannot assess SMS text messaging participation at all outside of a participant’s report; two-way messaging is still not definitive, as patients may see and not respond to the reminders, leading to reliance on the self-report as well.

#### User Preferences

Studies exploring participant preferences examined themes and topics of interest, as well as the frequency and timing of messages. Themes of interest to patients included messages about the following: mental health symptoms, treatment and management (eg, medication and appointments), lifestyle behaviors (eg, exercise, diet, and sleep), social relationships and leisure activity, motivation and goal setting, and independent living [43]. Another study examining personalized SMS text messaging reminders found the most requested reminders were for medication, appointments, and physical health (eg, exercise and diet) [44]. A survey study found that patients were interested in learning about a variety of psychoeducational topics including medication and side effects via digital platforms such as SMS text messaging [55].

Kauppi et al [44] examined trends in operational preferences, reporting that the preferred average number of texts per month was 10, with a preference for delivery early in the week (eg, Monday or Tuesday) and in the morning (eg, between 6 AM and noon). However, preferences differed according to a number of demographic factors such as age, gender, marital status, living situation, and age at first contact with treatment.

## Discussion

Overall findings support the use of SMS text messaging as a means to enhance engagement in treatment-seeking individuals with psychosis. All but one study demonstrated that an SMS text messaging intervention was associated with improved clinical engagement. Moreover, all studies found that no significant harm was associated with SMS text messaging interventions. Similarly, feasibility findings suggest overall patient endorsement for the use of SMS text messaging in the treatment of psychosis.

### Medication

Using SMS text messaging for medication reminders appears to have a significant positive effect on both attitudes about medication [47] and medication adherence [47,50,54], with the potential to have lasting effects [49]. However, there are many caveats to these findings. First, findings, although positive and significant, had moderate effect sizes based on small samples, with some trials reporting nonsignificant changes or significant changes for only the subgroups of the study sample. Second, most studies did not investigate effects after the termination of the intervention, highlighting a need for additional longitudinal follow-up studies.

It is also important to acknowledge that medication reminders only address involuntary nonadherence (ie, motivation, forgetfulness, and understanding) and do not directly address voluntary nonadherence (ie, lack of willingness) [57] except potentially indirectly through participants' attitude toward medication. As a cautionary note, this should be considered when evaluating medication reminders as different causes of nonadherence may require different approaches. Furthermore, results indicate SMS text messaging reminders to be most efficacious for patients in a midrange of symptom severity and functioning [47,52,54].

The study that did not find SMS text messaging reminders to have a significant impact on medication adherence used hospital admission rates as the primary outcome [46]. Despite reductions in hospital admissions being one goal of treatment, decreases in hospitalization rates as an indicator of improved medication adherence may be too far removed from the adherence process and may not be an appropriate indicator of the efficacy of SMS text messaging reminders.

The results shown here are similar to those across other areas of health care such that many show positive trends toward improved treatment adherence, with mixed results with regard to significance. For example, some studies find a positive nonsignificant trend toward improved medication adherence in postsurgery populations [58] and individuals with diabetes [33]. Other studies report strong findings in areas such as HIV antiretroviral treatment [32] and general health populations [59]. In contrast, some studies have found no effect of SMS text messaging reminders on populations such as patients with tuberculosis [60].

Some of the aforementioned studies [33,58] as well as many in this review used one-way messaging, which may explain some of the nonsignificant findings. A meta-analysis examining one-way versus two-way SMS text messages as medication reminders found that two-way messaging can increase oral medication adherence in a breadth of medical conditions by 23%, whereas one-way messaging shows little or no effect [61]. In this review, 6 interventions used one-way SMS text messaging reminders, yet only 2 studies found nonsignificant changes in either attendance or medication adherence; thus, one-way reminders may still be effective in psychosis populations.

### Attendance

Regarding clinic attendance, SMS text messaging reminders were also found to increase the likelihood of attending appointments [51,52]. The target population in the study by Thomas et al [51] was patients who had not yet had contact with psychosis services, yet the use of SMS text messaging reminders increased the likelihood of clinic attendance two-fold at initial appointments. Similar findings are reported for initial attendance at treatment start in substance use literature [62]. This is notable considering the high rates of disengagement and dropout for patients in early stages of accessing care [14] and, therefore, represent a population at elevated risk of not receiving treatment.

Findings in this review replicate previous positive findings on attendance within various health conditions and settings, including primary care, mental health care, and dental care, where reminders improve attendance and decrease the probability of missed appointments [63-65]. Taken together, the positive effects of SMS text messaging on clinic attendance may have the potential for annual national cost-savings in the millions [66], and large health insurance providers have begun to consider reimbursement for use of digital aids [31].

### Therapeutic Rapport

Therapeutic rapport may also be enriched by the addition of SMS text messaging as patients in 1 included study reported improved therapeutic rapport with SMS text messaging interventionists compared with their clinical teams [42]. This may be due to factors relating to perceived availability of the mobile interventionist compared with the clinical team [42], as the mobile interventionist is able to reply to questions and problems in real time and communicate with patients daily. This is an important finding given that therapeutic rapport is important for sustained engagement [14,15].

### Feasibility

Feasibility outcomes were assessed by both RCTs and qualitative studies, providing important insights for future implementation. All studies examining user outcomes found SMS text messaging to be feasible, safe, and acceptable, gaining the majority endorsement in each sample [42,44,54-56]. These findings help assuage popular concerns around the feasibility of SMS text messaging interventions as cell phone ownership [55,56] and response rates were high [42,54], most were familiar with SMS text messaging or found it easy to learn and use [49,52,56], and only 1 patient reported increased paranoia around usage of mobile phones [56].

In comparison with other mobile strategies such as mobile apps, SMS text messaging studies reported slightly higher response/usage rates (83%-87%) than mobile app studies (69%-86%) in this population [29], although attrition rates were similar [29]. In a direct comparison of SMS text messaging and mobile apps for daily symptom monitoring, authors noted that the set up used for apps is a more involved process and may affect participation rate; however, the user platform in apps was preferred over SMS text messaging by participants [67].

A clear advantage of SMS text messaging over mobile apps is cell phone versus smartphone ownership, and concerns around the exclusion of economically disadvantaged populations, such as those reliant on government financial support. Cell phone ownership steadily decreases with income and is significantly lower among individuals with severe mental illness (SMI). Ownership among earners of an annual income under US $30,000 per year is 84% and 81% for those with psychosis, yet ownership among the lowest earners drops to 50% and 35%, respectively [29]. Cell phone ownership among SMI populations has been reported at 93% in the United States, yet only 50% to 60% owned smartphones [68,69], and 67.9% in Israel [70]. In developing and newly developed countries such as India, however, smartphone ownership is still only present in a minority (22.7%) of mental health populations [71]. Importantly, trends in smartphone ownership, although still below cell phone ownership rates as of 2018, have been steadily increasing for years in the general population [72] as well as populations with psychosis [29] and will likely become more ubiquitous as younger populations grow in this digital age.

### Strengths and Limitations

A strength of this review is that we took a comprehensive perspective to engagement and provided an inclusive review of clinical engagement in populations with psychosis [14]. However, an even more comprehensive examination of technologically aided engagement could have been created by including other platforms that use mobile devices, such as mobile apps, as a helpful comparison group. This would help further elucidate patient preferences and effectiveness to ascertain information on which platform is best suited for implementation in the treatment of psychosis. Another limitation of this review may be the design itself, as a review is limited to data extracted from the published articles of the included studies, without attempts to contact corresponding authors of the included studies to obtain further information. In addition, this review was designed and registered as a qualitative review rather than a meta-analysis, although we acknowledge that a meta-analysis may have provided some more quantitative insights into the use of SMS text messaging to boost engagement in the treatment of psychosis; the meta-analysis itself would be limited given the small number of published studies and the significant heterogeneity in methods, primary outcomes, and durations of interventions..

Limited conclusions can be drawn from included studies owing to the scarcity of effectiveness trials and novelty of digital strategies in the treatment of psychosis. Additionally, many current trials are pilot studies that are time-limited with small sample sizes using convenience sampling and, in some cases, with underpowered analyses. Another limitation is that no study examined impacts on clinician workflow or cost-effectiveness or privacy considerations beyond educating participants on privacy risks. Furthermore, there are no standard measurements for clinical engagement or any of its domains, making results too heterogeneous to be accurately compared.

### Future Directions

The central theme drawn from the presented studies is that individuals with psychosis prefer a person-centered approach, with a personal feel, and that this approach tends to be more effective. Suggestions for future trials using SMS text messaging include the involvement of participants in co-design, employing two-way messaging to engage participants in a conversation and allowing for personal choice and autonomy within the intervention, namely personal choice over content, timing, and frequency. This is especially important given the considerable heterogeneity in psychosis spectrum disorders and the flexibility of SMS text messaging to afford opportunities for individualized treatment approaches. Moreover, digital tools have the potential to increase patient autonomy and independence by extending care outside the clinic and putting it literally in patients’ hands.

Given the heterogeneity of clinical presentation and treatment, it should also be considered that not all patients with psychosis are prescribed medication, so standalone medication reminders would not be universally helpful. Other subgroups requiring additional planning include individuals with physical disabilities, low literacy levels, severe cognitive impairments, developmental delays, experiencing homelessness, or share a cell phone with others [42]. Another key area of consideration is safety protocols. Despite a study’s report that several urgent issues had to be escalated to the primary care team resulting in expedited prescription fills, home visits, and safety assessments [43], none of the included studies reference precautions or procedures relating to safety. Such issues underscore the importance of developing safety protocols for digital tools.

Finally, due to the potential increase in patient autonomy and independence, SMS text messaging–augmented care could lead to a decreased dependence on clinical staff, allowing clinicians more flexibility to manage caseloads and potentially leading to positive financial and economic outcomes while simultaneously allowing for better, more personalized care. Future studies should, therefore, report on the cost-effectiveness of the interventions as well as impact on workflow to assess how readily SMS text messaging can be implemented.

### Conclusions

In sum, this review demonstrates the potential of SMS text messaging to be used as an adjunct platform to support clinical treatment as a means of improved engagement. These findings show that SMS text messaging is extremely well tolerated, safe, and accepted among individuals with psychosis, testifying to its potential to not only improve engagement in care but also to extend care beyond the clinic. To this end, SMS text messaging stands to offer a pragmatic solution to boost clinical engagement and provide an alternative avenue to access treatment. To this end, a more thorough examination of practical considerations, consistent measures for engagement factors, and rigorous examinations of its outcomes (eg, medication blood plasma levels or electronic medication caps compared with self-reporting) are required to adequately assess its efficacy, cost-effectiveness, and readiness for implementation.

## Appendix

Multimedia Appendix 1 Summary of results for included studies.

## Abbreviations

RCT: randomized controlled trial
SMI: Severe Mental Illness
