# Supplementary material for: The Use of Text Messaging to Improve Clinical Engagement for Individuals With Psychosis: Systematic Review
Source: JMIR Ment Health. 2020 Apr 2;7(4):e16993. doi: 10.2196/16993 (PMC7163420; doi:10.2196/16993)
Supplement: Multimedia Appendix 1 [file mental_v7i4e16993_app1.docx]

**Table 5.** Summary of main results of included studies.

| References | | Primary outcome: engagement target | *P* value | Cohen *d* | Sufficient power? | | | Secondary outcome | *P* value | Additional findings | Attrition (%) | Values, n |
| --- | --- | --- | --- | --- | --- | --- | --- | --- | --- | --- | --- | --- |
| **Studies based on experimental outcomes** | | | | | | | | | | | | |
|  | Välimäki [48]^a^ | No change in the number of hospitalizations | - .28 | - Risk ratio 1.11 | Yes | | | - No differences found between groups in any secondary outcomes | - N/A^b^ | No differences found between groups in any secondary outcomes | 4.80 | 1119 |
|  | Montes et al [43] | Improved MedAd^c^ at 3 months | - .02 | - 0.33^d^ | Yes | | | - Improved treatment attitude and quality of life | - .0003 - .03 | Maintained MedAd at 6 months (*P*=.04) | 1 | 340 |
|  | Xu [46] | Increased MedAd by 27% | - .004 | - 0.35 | Yes | | | - Less loss of functioning | - .117 | 90% patient satisfaction | 4.30 | 237 |
|  | Menon et al [47] | Improved MedAd | - <.001 | - 0.76 ^d^ | Yes | | | - Maintained at 6 months - Improved treatment attitude - No change: quality of life/symptoms | - <.001 - .01 - >.05 | Involvement of a caregiver may explain some of the success | 4.70 | 278 |
|  | Beebe et al [45] | Comparison between phone (81%) and SMS text messaging (71.5%) and phone and SMS text messaging (81%) | - NC^e^ - NC | - .45^d^ - .36 ^d^ | No (34%) | | | - Improved MedAd and symptoms in the phone and SMS text messaging group | - .31 | Notable: depot medications were included | 20 | 30 |
|  | Thomas et al [50] | SMS text messaging predicted attendance | - .045^d^ | - 0.32^d^ | Yes | | | - No Vx predicted appointment attendance | N/A | No SMS text message receipt | NR^f^ | 113 |
|  | Montel et al [43] | Improved appointment attendance by 26% and MedAd by 8% | - <.05 - <.05 | - NC - NC | Yes | | | - Improved symptoms only among SMS text message responders | - .02 | 70% positive review, 41% effective, and 47% continued | 13 | 62 |
|  | Kraviriti et al [51] | Improved appointment attendance | - .001 | - 0.59 ^b^ | Yes | | | - Odds ratio of 2.95 for appointment attendance | N/A | N/A | N/A | 75 |
|  | Granhoml et al [44] | Improved MedAd | - <.001 - <.001 | - −1.40 ^d^ - −2.74 ^d^ | Yes | | | - No change in overall symptom scores | N/A | Response rate: 83%-86% | 0 | 55 |
| **Studies based on feasibility outcomes** | | | | | | | | | | | | |
|  | Ben-Zeev et al [52]^a^ | Improved treatment alliance | - .01 | - NC | | Yes | | - Feasibility findings: 90% reported satisfaction, 87% found it to work, 80% said it was easy to learn, and 90% found it useful and fun. Response rate: 87% | N/A | N/A | 6 | 28 |
|  | Aschbrenner et al [55]^a^ | Qualitative findings: 7 themes: symptoms, lifestyle, coping, social/leisure, motivation, and independent living | N/A | N/A | | N/A | | N/A | N/A | N/A | N/A | 17 |
|  | Lal et al [54] | Preferred platform for reminders: SMS text messaging | N/A | N/A | | N/A | | - Most popular topic: medication | N/A | 6% did not own a phone | N/A | 403 |
|  | Bogart et al [49] | Feasibility findings: 82% owned a phone, 80% knew how to use SMS text messages, and 59% wanted the reminders | N/A | N/A | | N/A | | - Other findings: reasons for overall nonadherence—intentional missed dose=49%, unintentional missed dose=35% | N/A | N/A | N/A | 85 |
|  | Kauppi et al [56]^a^ | 98% easy to use, 87% caused no harm, 72% satisfied, 61% useful, and 64% wanted to continue | N/A | N/A | | | N/A | - 13% said it did cause harm (eg, woke them up, annoying, and disrupting work). Response rates differed by demographics. | N/A | N/A | N/A | 67 |
|  | Kannisto et al [53]^a^ | Most commonly chosen reminders were for medication, appointments, and exercise | N/A | N/A | | | N/A | - Timing: start of week and in the morning. Preferences differed by demographic variables | N/A | N/A | 4 | 562 |

^a^Indicates a shared sample.

^b^N/A: not applicable.

^c^MedAd: medication adherence.

^d^Indicates that the effect size was calculated by the review study team, while no star indicates the effect size was reported in the original paper.

^e^NC: not calculable.

^f^NR: not reported.
